# Interfacial and colloidal drivers of wine drying astringency: the role of wine–saliva aggregates^☆^

**DOI:** 10.1016/j.fochx.2026.104223

**Published:** 2026-07-26

**Authors:** Emerson Núñez, Edmundo Bordeu, Max Marian, Martin Vrbka, David Nečas, Fernando Osorio, Andreas Rosenkranz, V. Felipe Laurie, Natalia Brossard

**Affiliations:** a Department of Fruit Production and Enology, School of Agricultural Science and Natural Systems, Pontificia Universidad Católica De Chile, Av. Vicuña Mackenna 4860, Macul, Chile; b VitiScience Research and Innovation Center - CIA 250013, School of Agricultural Science and Natural Systems, Pontificia Universidad Católica De Chile, Av. Vicuña Mackenna 4860, Macul, Chile; c Institute of Machine Design and Tribology (IMKT), Leibniz University Hannover, An Der Universität 1, Garbsen 30823, Germany; d Department of Mechanical and Metallurgical Engineering, School of Engineering, Pontificia Universidad Católica De Chile, 6904411 Macul, Chile; e Department of Tribology, Faculty of Mechanical Engineering, Brno University of Technology, Technická 2896/2, 616 69 Brno, Czech Republic; f Department of Food Science and Technology, Technological Faculty, Universidad De Santiago De Chile. Av. El Belloto 3735, Estación Central, Santiago, Chile; g Department for Chemical Engineering, Biotechnology and Materials, University of Chile, Santiago, Chile; h Enology Laboratory, School of Agricultural Sciences, Universidad De Talca, Campus Talca, Chile.

**Keywords:** Oral tribology, Astringency, Red wine, Tannin–protein aggregates, Film thickness, Human saliva

## Abstract

Astringency in red wine is associated with lubrication changes driven by tannin–saliva interactions, yet the physicochemical basis of astringency subqualities remains unclear. Focusing on drying, a key quality-related subquality, we examined whether wine–saliva aggregates can contribute to lubrication stability rather than only increasing friction. Red wines spanning non-drying to highly drying profiles were mixed 1:1 with unstimulated human saliva. Film thickness and friction were measured under controlled, oral-inspired sliding conditions, and mixtures were centrifuged to obtain soluble (supernatant) and insoluble (pellet) fractions characterized for colloidal stability, interfacial wetting, turbidity, viscosity, and macromolecular composition. Lower drying was associated with thicker films and lower friction, with more stable, entrainable supernatants showing more negative zeta potential, smaller colloids, higher suspended turbidity, and greater polysaccharide support. Overall, the results support a supernatant-centric view in which aggregate functionality and morphology relate to drying subquality, suggesting routes to soften mouthfeel without necessarily reducing phenolics.

## Graphical abstract

**Figure:**
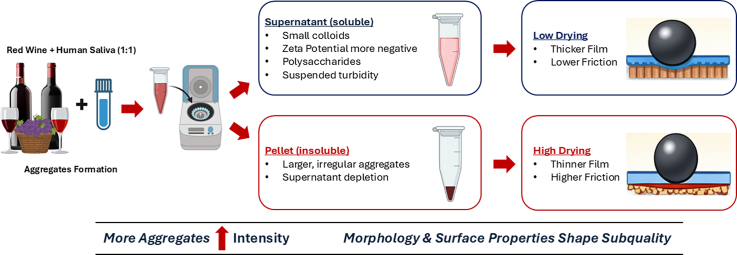

## 1 Introduction

Mouthfeel attributes are key determinants of wine sensory quality, shaping organoleptic perception and ultimately driving consumer acceptance (Wang et al., 2024; Williamson et al., 2012). From an enological standpoint, the management of grape astringency must therefore be considered during harvest decisions and throughout vinification to optimize quality potential, particularly in cultivars characterized by elevated or difficult-to-modulate astringency arising from complex tannin–salivary protein interactions that strongly influence oral tribological responses and perceived mouthfeel (Brossard et al., 2021; Rousseau & Delteil, 2000; Rudge et al., 2019).

Astringency, a central contributor to wine mouthfeel, is typically described as sensations of dryness, roughness, and oral contraction, often attributed to increased frictional forces between oral surfaces following interactions between wine polyphenols and salivary proteins (Brossard et al., 2021; Upadhyay et al., 2016). Nevertheless, the underlying mechanisms remain debated, particularly regarding the formation, structure, and behavior of tannin–protein aggregates in the oral cavity. Several studies have linked astringency to lubrication loss caused by precipitation of complexes between red wine tannins and salivary proline-rich proteins (PRPs) (Brossard et al., 2016; Zhu et al., 2024), whereas others propose that tannins disrupt salivary pellicles or interact with oral epithelial membranes, exposing mechanoreceptors or activating chemosensory pathways that contribute to astringency perception (Liu et al., 2023; Ma et al., 2014; Reis et al., 2020; Rosenkranz et al., 2021). Astringency is not perceived as a single sensory modality, but as a multidimensional experience that includes subqualities such as softness, adhesiveness, dryness, and grip, which vary across grape cultivars and wine matrices (Paissoni et al., 2023; Pickering & Demiglio, 2008; Wang et al., 2024). Among these subqualities, drying is especially relevant because it refers to feelings of lack of lubrication or desiccation in the mouth, according to the mouth-feel terminology proposed for red wines by Gawel et al. (2000), and has been reported to correlate strongly with tannin-related variation in red wines (Pavéz et al., 2022). This subquality is particularly suitable for physicochemical and tribological study because it can be linked more directly to salivary film depletion, impaired wetting, and frictional changes than broader or more hedonic descriptors. For this reason, the present work focused on drying as a quality-relevant astringency subquality that provides a tractable bridge between sensory categorization and lubrication-related oral mechanisms. This focus does not imply that other astringency subqualities are less important, but rather that drying offers the clearest sensory counterpart to the lubrication-related mechanisms evaluated in the present study.

This complexity complicates the interpretation of astringency as a single sensory attribute, as different subqualities may arise from distinct molecular, colloidal, and tribological mechanisms. For example, differences in tannin size, structure, galloylation degree, and interaction with anthocyanins have been shown to influence astringency intensity and quality (Vidal, Francis, Williams, et al., 2004; Ferrero-del-Teso et al., 2022; Zhao et al., 2023). In this context, previous work has suggested that aggregate formation may contribute to differences in oral lubrication and astringency subquality. For example, wines perceived as softer or less drying have been reported to produce fewer precipitated tannin–salivary protein aggregates than more drying wines (Brossard et al., 2016; González-Muñoz et al., 2021). However, such comparisons should be interpreted cautiously, because aggregate abundance may also be influenced by matrix effects. In the comparative study of Carménère and Cabernet Sauvignon reported by Brossard et al. (2016), the wines differed not only in aggregate precipitation behavior but also in total tannin concentration and pH, and tannin structural features were not characterized. Thus, aggregate abundance alone cannot be considered independently of variables such as pH, ethanol, polysaccharides, anthocyanins, and tannin molecular architecture, all of which may influence the balance between soluble and insoluble complexes and, consequently, lubrication loss and frictional behavior (González-Muñoz et al., 2021; Kuhlman et al., 2024; McRae & Kennedy, 2011).

Pradal and Stokes (2016) described three possible lubrication outcomes when food systems interact with a pre-adsorbed salivary film: no interaction, interaction that reduces lubrication and increases friction, and synergistic interaction that enhances lubrication through muco-adhesive effects. Most research on tannin–protein systems has focused on the first two possibilities. In these cases, either precipitation-driven roughness or non-precipitating molecular interactions disrupt lubrication, leading to increased friction and perceived dryness. By contrast, the possibility that specific tannin–protein complexes could behave as tribologically active, muco-adhesive structures capable of stabilizing or even enhancing oral lubrication has not been systematically explored (Agorastos et al., 2022). Such a mechanism could help explain why some wines with high tannin content are still perceived as smoother and less drying, and it is also consistent with empirical practices such as protein fining, which reduce perceived astringency through controlled tannin–protein interactions.

These observations suggest that differences in astringency subqualities among grape cultivars may arise from the balance between aggregate abundance and functionality, in which tannin-related molecular features and wine-matrix composition influence the formation and interfacial behavior of wine–saliva complexes, thereby increasing or decreasing friction depending on their physicochemical properties. Because tannin structural features were not specifically characterized in the present study, this aspect is considered here as a plausible mechanistic contributor rather than as a directly measured variable. Understanding when wine–saliva complexes behave as friction-enhancing particles rather than lubrication-enhancing networks may therefore represent a missing link between colloidal chemistry, oral tribology, and the sensory perception of wine mouthfeel. Based on this framework, the present study applies complementary physicochemical and tribological approaches to achieve a multidimensional understanding of astringency mechanisms and the behavior of tannin–protein complexes within the oral environment. Red wines with varying astringency intensities and drying subqualities were evaluated to elucidate how aggregate properties and lubrication behavior contribute to perceived mouthfeel.

## 2 Materials and methods

### 2.1 Samples

#### 2.1.1 Red wines

The red wine samples came from different production valleys in Chile's central wine-growing region (Maipo, Cachapoal, and Maule). The set included Carménère, Carignan, and Cabernet Sauvignon wines selected to represent contrasting drying-related mouthfeel profiles, as detailed in Table 1.

**Table 1:** Red wine samples by origin and astringency subquality.

Variety	Area	Valley	Year	Sensory Astringency^a^	Code
Carménère (CMR)	Peumo	Cachapoal	2024	No Drying (Smooth) – ND	CMR:ND
Carignan (CG)	Loncomilla	Maule	2024	Low Drying – LD	CG:LD
Carignan (CG)	Maipo	Maipo	2024	High Drying – HD	CG:HD
Cabernet Sauvignon (CS)	Maipo	Maipo	2024	Low Drying – LD	CS:LD
Cabernet Sauvignon (CS)	Maipo Alto	Maipo	2024	Medium Drying – MD	CS:MD
Cabernet Sauvignon (CS)	Curicó	Maule	2023	High Drying – HD	CS:HD

a Sensory classification was used only as a sample-selection criterion and was not intended as a formal quantitative sensory dataset. Wines were selected to represent contrasting drying-related mouthfeel profiles based on internal evaluations by trained winery enological staff, using professional tasting practices focused on drying as the primary descriptor. Selection was additionally supported by cultivar–valley typicity and previous literature describing differences in phenolic composition, mouthfeel expression, and wine–saliva aggregate behavior in Chilean red wines, particularly for Cabernet Sauvignon, Carménère, and Carignan from recognized Chilean viticultural areas (Brossard et al., 2021; Cáceres-Mella et al., 2014; Gutiérrez-Gamboa and Moreno-Simunovic, 2018, Gutiérrez-Gamboa and Moreno-Simunovic, 2019; Martínez-Gil et al., 2018).

The wine set was designed to cover a broad and commercially relevant range of drying expression, rather than to provide a formal sensory classification of all astringency subqualities. Therefore, wine selection combined expert enological screening with cultivar–valley typicity and literature-supported expectations regarding phenolic composition and mouthfeel. Cabernet Sauvignon from Maipo/Alto Maipo was included because this cultivar and origin are recognized for structured red wines with relevant phenolic and tannin expression, making them suitable for representing medium-to-high drying profiles. This is consistent with previous studies showing that Cabernet Sauvignon and Carménère can differ in astringency subquality and wine–saliva aggregate behavior, with Cabernet Sauvignon associated with rougher astringency and Carménère with softer or less drying mouthfeel expression (Brossard et al., 2021). Moreover, studies on Chilean Cabernet Sauvignon blends have shown that varietal matrix composition can modify phenolic composition, proanthocyanidin profile, and mouthfeel characteristics, reinforcing the importance of cultivar and matrix effects in the interpretation of oral perception (Cáceres-Mella et al., 2014).

Carignan from Maule/Loncomilla was selected because this cultivar and region have been associated with distinctive phenolic composition, terroir expression, and renewed oenological relevance in Chilean wines (Gil and Pszczólkowski, 2015). Previous studies have shown that location within Maule Valley affects ripening behavior and grape phenolic composition in Carignan vineyards, supporting its inclusion as a contrasting phenolic and mouthfeel matrix (Gutiérrez-Gamboa and Moreno-Simunovic, 2018, Gutiérrez-Gamboa and Moreno-Simunovic, 2019; Martínez-Gil et al., 2018). Carménère from Peumo/Cachapoal was included as a smooth/non-drying anchor, consistent with previous evidence linking Carménère to softer astringency expression and distinctive wine–saliva aggregate characteristics compared with Cabernet Sauvignon (Brossard et al., 2021, Jara et al., 2017)).

Thus, the wine set was defined using convergent criteria: expert enological screening, cultivar–valley typicity, and previous literature on phenolic composition, mouthfeel, and wine–saliva aggregate behavior. Sensory information was used only for sample selection and not as a stand-alone descriptive sensory dataset or formal sensory validation of all astringency subqualities.

#### 2.1.2 Human saliva (HS)

Unstimulated HS was used because of its higher interaction capacity with wine polyphenols compared with stimulated saliva and its lower water content (Brossard et al., 2016; Rinaldi et al., 2012). Saliva was collected from 20 healthy, non-smoking volunteers recruited at the San Joaquín campus of the Pontificia Universidad Católica de Chile. Participants were instructed to refrain from eating and drinking (except water) for 2 h before collection. Collection was performed in the afternoon to reduce circadian variation in salivary composition, in line with previous protocols reported by Brossard, Cai, Osorio, Bordeu and Chen, 2016, Brossard et al., 2021. Subjects passively accumulated saliva in the mouth and expectorated it into clean centrifuge tubes over 15 min, with bottled water allowed between collection intervals to facilitate saliva recovery. Immediately after collection, saliva from all donors was pooled and centrifuged at 500 ×*g* for 10 min to remove suspended debris using a Sorvall® ST-21 centrifuge (DuPont, Newtown, CT, USA). The clarified supernatant was dispensed into 15 mL tubes and stored at −80 °C until use. Before experiments, the required aliquots were thawed and equilibrated to 28 °C in a water bath to simulate oral surface temperature (Engelen & de Wijk, 2012). No separate preliminary stability assay was performed. Instead, compositional changes were minimized operationally by immediate pooling and centrifugation, aliquot storage at −80 °C, equilibration to 28 °C only immediately before use, and immediate analysis of wine–HS fractions after mixing and phase separation. Saliva collection sessions were scheduled in close temporal proximity to the corresponding experimental runs in order to minimize storage time prior to use. All procedures were explained to participants, informed consent was obtained before each collection session, and the study was approved by the Scientific Ethics Committee of the Pontificia Universidad Católica de Chile (ID: 240911005).

#### 2.1.3 Wine and saliva mixtures

Because wine polyphenols readily interact with salivary proteins to form soluble and insoluble tannin–protein complexes/aggregates — which can alter oral lubrication and modulate astringency (Brossard et al., 2016; Soares et al., 2017) — red wines were mixed 1:1 *v*/v with HS. A 1:1 (*v*/v) wine:HS ratio was selected as a standardized experimental condition to model the fluid mixture remaining in the mouth during or immediately after wine swallowing, while preserving comparability with previous tribological and wine–saliva aggregate studies from our group (Brossard, Cai, Osorio, Bordeu and Chen, 2016, Brossard et al., 2021). We do not claim that this proportion exactly reproduces the in vivo oral environment, where saliva-to-wine ratios are dynamic and likely depend on sip volume, salivary flow, and oral processing. Rather, it was used as a controlled approximation that allows reproducible comparison of colloidal, interfacial, and tribological responses across wines. This limitation is considered when relating instrumental data to sensory drying. For each wine–saliva combination (per grape variety), the insoluble and soluble aggregate fractions were separated by centrifugation (500 ×*g*, 10 min) within 30 s of mixing, without an additional incubation step, yielding a pellet (insoluble aggregates) and a supernatant (soluble fraction), respectively (Brossard et al., 2021). Both fractions were retained for downstream characterization and analyzed by complementary physicochemical and tribological methods whenever feasible. Given its liquid nature and lower particulate content, the supernatant was generally more amenable to a comprehensive analytical panel. In contrast, methods applicable to the pellet were selected based on practicality and sample-handling constraints. All methods that used supernatant and precipitate samples were performed immediately after pooling and centrifugation to standardize run times. The pellet fraction was therefore handled as a wet insoluble phase and gently homogenized only as required for the applicable measurements, without attempting complete redissolution or dissociation of the aggregates. Accordingly, pellet-related data should be interpreted as an operational characterization of the insoluble fraction and of the material transferred out of the lubricating phase, rather than as a full molecular characterization of protein–tannin interactions within that phase.

### 2.2 Wine compositional analysis

The wines were characterized in terms of their elemental chemical composition (e.g., alcohol, titratable acidity, volatile acidity, pH, residual sugars, and free and total sulfur dioxide) using standard methodologies (Bordeu & Scarpa, 1998). The phenolic compounds were analyzed using the total polyphenol index (TPI), color intensity (CI), hue, and total anthocyanin content (TAC), all measured in triplicate, according to standard methodology (Bordeu & Scarpa, 1998; Ribéreau-Gayon et al., 1976). These baseline compositional analyses were performed on the neat wines to provide the chemical context for the subsequent interpretation of wine–saliva interactions, supernatant behavior, and tribological responses. Tannin content was determined using the MCP (methyl cellulose precipitable) tannin assay, following the fact sheet provided by The Australian Wine Research Institute (2015) and previous methodological work on MCP application in wine matrices (Mercurio et al., 2007).

#### 2.2.1 Protein and polysaccharide content determination

Colorimetric assays measured protein and polysaccharide contents. To minimize interference from wine phenolics, samples were first treated with polyvinylpolypyrrolidone (PVPP; 5 mg/mL, Merck Millipore, MA, USA). Suspensions were agitated on an orbital shaker for 1 h, and PVPP was removed by centrifugation (3500 ×*g*, 5 min, 4 °C, DuPont, Sorvall® ST-21, Newtown, CT, USA). The resulting supernatants were filtered through 0.45 μm PES syringe filters (Ø 22 mm, Biocomma, Guangdong, China) and used immediately for analysis. This workflow was applied to wines and, similarly, to the supernatants of 1:1 *v*/v wine–HS mixtures. This filtration step was applied only to samples intended for the colorimetric protein and polysaccharide assays described in this section, after PVPP treatment and clarification. It was not applied to the native supernatants used for psize, ζ-potential, ratio determination, or turbidity measurements, which were analyzed after centrifugation without membrane filtration.

A Bradford assay for protein quantification was performed after acetone/trichloroacetic acid precipitation. Briefly, 500 μL of PVPP-treated sample was mixed with 1.0 mL ice-cold acetone (Winkler, Santiago, Chile) containing 10% *w*/*v* TCA (Merck Millipore, MA, USA). Mixtures were held at −18 °C for 16 h and centrifuged (14,000 ×*g*, 15 min, 4 °C). Pellets were washed once with 1.0 mL acetone, vortexed, and centrifuged again (14,000 *g*, 10 min, 4 °C). Open tubes were placed on a heating concentrator at 65 °C for 30 min (NB-503GBP, N-Biotek.INC., Bucheon-si, Korea) to dry the pellets. Pellets were re-dissolved in 500 μL distilled water; 100 μL aliquots were combined with 1.0 mL Bradford reagent (Merck Millipore, MA, USA), mixed, and read at 595 nm on a UV-VIS spectrophotometer Metertech SP-8001 (Metertech Inc., Taipei, Taiwan). Bovine serum albumin (BSA; 0–1000 mg/L; Sigma-Aldrich) was used to construct the calibration curve.

For polysaccharide quantification, an ethanol precipitation was followed by the phenol–sulfuric acid reaction. In short, 20 μL of filtered, PVPP-treated sample was combined with 500 μL absolute ethanol (Supelco, Merck Millipore, MA, USA), stored at 4 °C for 16 h, and centrifuged (14,000 ×*g*, 30 min). Pellets were dried on a 65 °C heating concentrator for 30 min (NB-503GBP, N-Biotek, INC., Bucheon-si, Korea) and then dissolved in 1.0 mL of a water/phenol solution (2% *v*/v phenol in water; Merck Millipore, MA, USA). Subsequently, 400 μL of this solution was transferred to a clean tube, and 1.0 mL concentrated sulfuric acid (Winkler, Santiago, Chile) was added. After 30 min at room temperature, absorbance was measured at 490 nm. Quantification was performed using a glucose standard curve (0–1000 mg/L; Sigma-Aldrich) in the same water/phenol solution, which produces a colorimetric reaction. Because wine and wine–HS mixtures are chemically complex colloidal systems, the protein and polysaccharide values obtained here should be interpreted as operational, method-dependent estimates rather than as exhaustive compositional measurements. In particular, PVPP pretreatment was used to reduce phenolic interference in the colorimetric assays, but it may also remove polyphenols and associated macromolecular species, including protein–tannin and potentially polysaccharide-associated complexes. As a result, the absolute values may underestimate the total amount of bound macromolecular material originally present in the sample. Nevertheless, because the same pretreatment and analytical workflow were applied consistently to all wines and supernatants, these data remain useful for comparative interpretation across samples (Marangon et al., 2025; Marassi et al., 2021).

### 2.3 Physical and morphological characterization

#### 2.3.1 Ratio, psize and zeta potential (ζ)

First, the wine was mixed with HS (1:1) to determine the ratio of precipitated aggregates to supernatant by centrifugation at 500 ×*g* for 10 min. Then, the supernatant was separated from the aggregates and weighed to determine the respective percentages. The hydrodynamic diameter, the size distribution percentile d_0.9_, and the ζ-potential of the suspended particles in the supernatant of the mixtures were measured using the dynamic light scattering (DLS) technique on the Litesizer DLS 500 (Anton Paar, Graz, Austria). The aggregate detection considered the refractive indices of red wine and saliva (1:1) (1.338) and of water, used as a dispersant (1.33). All measurements were performed in triplicate.

#### 2.3.2 Contact angle and surface tension

Contact angle (CA) measurements were conducted using the sessile drop method at room temperature in a Drop Shape Analyzer (Krüss, Hamburg, Germany). Drops of 2 μL from wine and their mixtures with HS (1:1) were produced using a syringe (Injekt®-F Luer Solo) and placed on the polydimethylsiloxane surface (PDMS). The drops were illuminated by a high-power monochromatic LED and captured by a high-speed camera (DSA25, Krüss). Surface tension (SFT) was assessed by analyzing the shape of a liquid drop suspended from the tip of a needle (pendant drop technique), using a 1 mL syringe needle (Injekt®-F Luer Solo and DSA25, Krüss) (Ramteke et al., 2025). Five measurements were performed for each sample and mixture.

#### 2.3.3 Morphology analysis

Microstructure features of saliva and wine mixtures were observed using light microscopy (LM) to provide visual confirmation of the microstructure of the precipitated aggregates (Brossard et al., 2016). Each red wine was mixed with HS at a 1:1 ratio. The sample was placed on a slide immediately after mixing for microstructural observation. Photomicrographs were taken using a Primostar 3 microscope (Zeiss, Suzhou, China) equipped with an Axiocam 208 color digital camera (Zeiss) and a 10×/0.25 magnification lens. At least five images were captured for each sample. Labscope v.3.0 (Zeiss, Jenna, Germany) was used to collect, analyze, and insert the scale bar into the images.

#### 2.3.4 Turbidity analysis

Turbidity was measured with a turbidimeter 2100Q (Hach Co., Ames, IA, USA) on pure wines and on supernatants from 1:1 *v*/v wine–HS mixtures, in triplicate. For wine–HS systems, turbidity was recorded immediately after mixing, centrifugation, and supernatant separation, using a standardized workflow so that the interval between mixing and measurement was kept as short and consistent as possible across samples. Thus, the reported values represent an early post-mixing state of the supernatant rather than a long-term sedimentation equilibrium. The assay monitored the turbidity attributable to tannin–protein complex formation and retention in the soluble/suspended fraction (Brossard et al., 2020; Horne et al., 2002).

#### 2.3.5 Rheological behavior

Shear viscosity and oscillatory frequency sweeps were measured using a Discovery DHR-2 rheometer (TA Instruments Ltd., New Castle, DE, USA) equipped with a cone-and-plate geometry (1.008°, 60 mm diameter, 27 μm gap) at 28 °C, following a previously reported protocol with minor modifications (Engelen & de Wijk, 2012; Monasterio & Osorio, 2024). Wine, supernatant, and precipitate fractions obtained from 1:1 wine–HS mixtures were equilibrated for 5 min before analysis, and a water seal was used to minimize evaporation. Steady shear measurements were performed from 0.1 to 300 s^−1^ and back, whereas oscillatory frequency sweeps were run from 0.1 to 100 rad/s within the linear viscoelastic region (0.1–0.2% strain). Because phase separation limited the available sample volume, rheological measurements were performed in duplicate and are therefore interpreted as supportive/exploratory rather than as a basis for strong statistical inference.

Time-dependent flow behavior was summarized using the relative thixotropic area (RTA), calculated from the hysteresis between the upward and downward flow curves, as previously proposed for comparing systems with different viscosities (Badia-Olmos et al., 2022; Covacevich et al., 2024). Flow and oscillatory data were fitted to linear or Ostwald–de Waele models, as appropriate.

#### 2.3.6 Tribological measurements

**Fig. 1:**
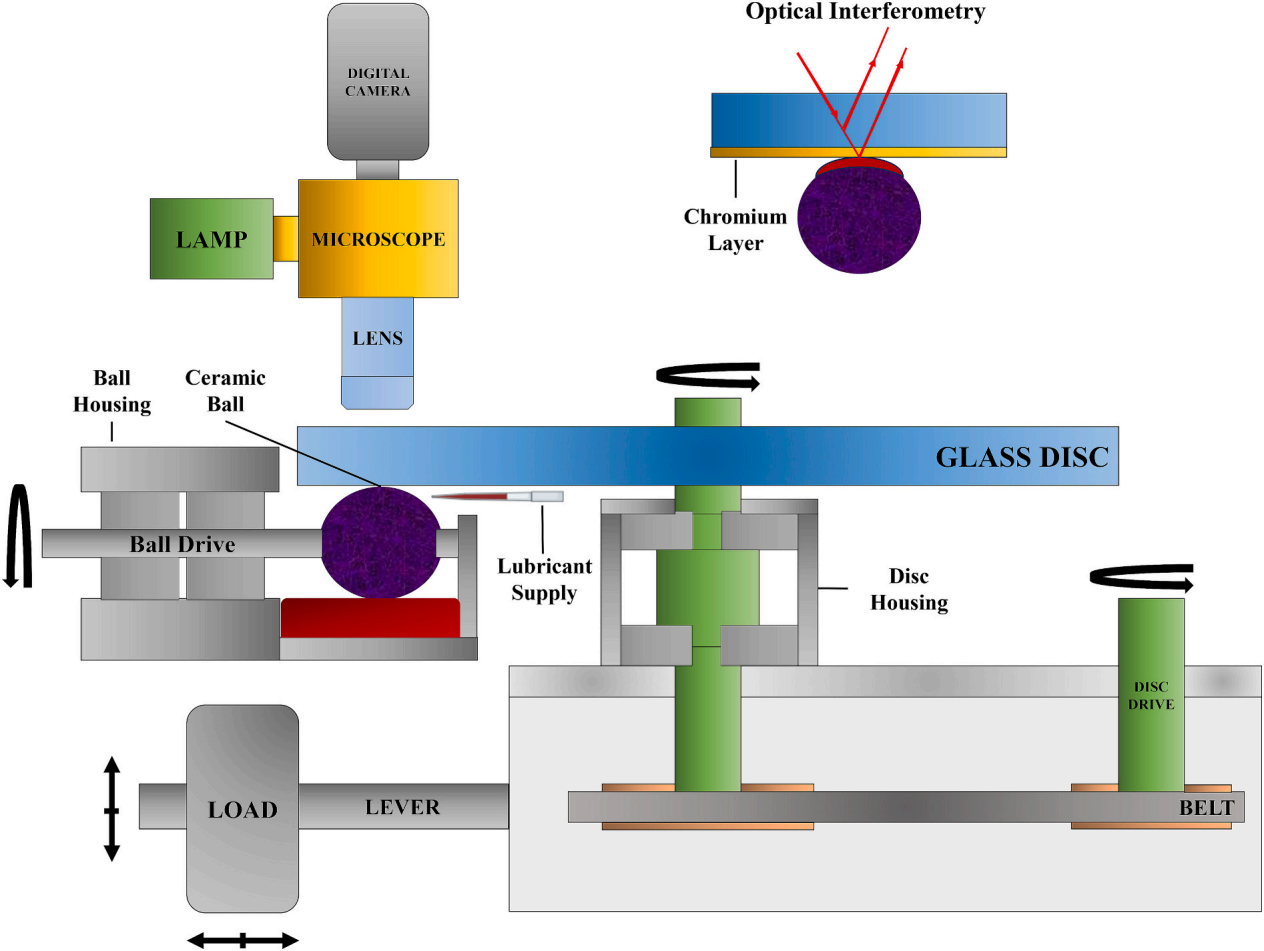
Scheme of the applied experimental approach. Modified from Nečas et al. (2016).

Tribological measurements of lubricant film thickness and friction were performed using a ball-on-disc tribometer with independently driven ball and disc components, allowing control of sliding conditions and slide-to-roll ratio (SRR). The experimental setup is shown in Fig. 1. The contact was monitored using an optical imaging system, and film thickness was determined by thin-film colorimetric interferometry following the general approach described by Nečas et al. (2016). In this method, interferograms are matched to a calibration curve to estimate the gap between the contacting surfaces.

The tribopair used in the experiments consisted of a ceramic ball with a nominal diameter of 25.2 mm and a glass disc coated with a thin chromium layer to enhance visibility of the interference. The initial surface topography of the components was examined using phase-shifting interferometry, employing a Bruker Contour GT-X instrument (Bruker, Billerica, MA, USA). Surface roughness (Ra) was measured at five locations within the contact area, yielding Ra = 30.5 ± 1.0 nm (mean ± SD, *n* = 5). The chromium-coated glass disc provided the reflective, optically smooth surface required for thin-film interferometry, producing stable interferograms across tests. Wine and its mixtures with HS (1:1) were used as a test lubricant. A lever system was employed to apply a load of 3 N. Film thickness (FT) and coefficient of friction (CoF) were recorded at 28 °C using forward and reverse speed sweeps (1–1000 mm·s^−1^) to identify the speed range in which film entrainment and friction responses were most stable and repeatable. Measurements were performed in triplicate. Based on this screening, all cross-sample comparisons and statistical analyses were performed within 150–750 mm·s^−1^, which captured the most consistent transition from boundary toward mixed lubrication. The tribopair materials were selected for chemical inertness and mechanical robustness under wine–saliva exposure, so that observed changes primarily reflect the lubricating film rather than substrate modification.

Carménère (CMR) serves as a low/non-drying anchor to frame both within- and between-variety trends, and principal component analyses (PCA) were configured to express deviations from this smooth/non-drying baseline. The normal load employed is higher than in much of the food-tribology literature; this leverages the instrument's capacity to stabilize film entrainment and improve signal-to-noise in the 150–750 mm·s^−1^ range. Although a higher load accentuates boundary contributions, the internal coherence between FT and CoF, as well as the qualitative ranking of samples, remained consistent; practically, discrimination was sharpened by suppressing spurious stick–slip (Shewan et al., 2020; Stokes et al., 2013).

### 2.4 Statistical analysis

Statistical analysis was conducted using STATGRAPHICS Centurion XVI, v.16.1.03 (StatGraphics Technologies, The Plains, VA, USA). Principal component analysis (PCA) was performed using JMP® 18.2.1 (853164) software (JMP Statistical Discovery LLC, Cary, NC, USA). Analysis of Variance (ANOVA) was utilized to assess the effects of wine and its mixtures with HS. Tukey's mean comparison was employed to identify significant differences among the treatments. PCA was applied as an exploratory visualization tool to examine relationships among selected wine compositional, supernatant, rheological, interfacial, and tribological variables. A significance level of *p* < 0.05 was maintained throughout the study.

## 3 Results and discussion

### 3.1 Physicochemical composition of the wines

The main physicochemical markers relevant to wine matrix characterization are compiled in Table S-1 (Supplementary), including turbidity, global phenolic indices (TPI, TTC, TAC), macromolecular indicators (polysaccharides and proteins), and matrix viscosity. Base enological parameters that contextualize matrix differences, such as alcohol, acidity, pH, residual sugars, color intensity, hue, and SO₂, are reported in Table S-2 (Supplementary). Together, these data describe the compositional background of the six wines and provide the basis for subsequent interpretation of wine–saliva interactions. Here, polysaccharide and protein values are interpreted as comparative macromolecular indicators derived from a common analytical workflow, rather than as exhaustive characterization of all colloidal macromolecules present in wine or wine–HS mixtures.

Across varieties, clear differences were observed in phenolic load, suspended turbidity, macromolecular composition, and viscosity. Carignan wines showed the highest turbidity values, whereas Cabernet Sauvignon MD and HD exhibited the highest polysaccharide contents. Protein content varied less among samples, while viscosity showed modest but significant differences across wines (Table S-1). From a compositional standpoint, these variables are relevant because polysaccharides, anthocyanins, and tannin-related matrix effects have all been associated with modulation of wine mouthfeel and tannin–protein interactions (Brandão et al., 2017; Ramos-Pineda et al., 2018; Vidal, Francis, Noble, et al., 2004; Vidal, Francis, Williams, et al., 2004; Watrelot & Norton, 2020).

Within Carignan, CG:LD exhibited higher TPI, TTC, anthocyanins, and color intensity than CG:HD, while both wines shared similarly low pH values (Tables S-1 and S-2). These differences indicate that, within the same cultivar, wines classified as low- and high-drying may still differ substantially in phenolic load and matrix composition. Within Cabernet Sauvignon, the low-to-high drying set spanned a broader compositional range: CS:MD showed the highest polysaccharide content, CS:HD the highest volatile acidity and residual sugar, and CS:LD the lowest viscosity. CMR exhibited intermediate phenolic values, moderate turbidity, and balanced macromolecular content, supporting its role as the non-drying/smooth reference sample in the present wine set.

Overall, the wines differed not only in global phenolic indices but also in turbidity, macromolecular composition, viscosity, and basic enological parameters, underscoring that drying contrasts were evaluated in chemically distinct wine matrices. In this context, the observed differences should not be interpreted solely as effects of total phenolic concentration, but rather as arising from compositional backgrounds that may differentially influence colloidal behavior and subsequent wine–saliva interactions, consistent with previous reports showing that basic wine composition also contributes to variability in astringency and dryness perception (Brandão et al., 2017; Pavéz et al., 2022; Vidal, Francis, Williams, et al., 2004; Watrelot & Norton, 2020).

### 3.2 Lubrication profiles

Film thickness and friction in the 150–750 mm·s^−1^ window are summarized in Table 2, enabling comparison across subqualities and varieties relative to CMR. Although the samples are organized according to drying classification, these responses should be interpreted in the context of the distinct compositional backgrounds described in Section 3.1, rather than as effects of drying level alone. The selected speed window captures a transition toward the mixed/hydrodynamic regime, where film thickness governs the reduction in friction. For Carignan wines, CG:LD formed a thicker film when mixed with HS than CG:HD, even though LD has more total tannins in the wine. This suggests that CG:LD generated more film-forming or more stable aggregates with HS than CG:HD. For CS, MD-HS achieved the highest film thickness, exceeding LD and HD. Although this effect cannot be attributed to a single compositional factor, its higher polysaccharide content and intermediate anthocyanin level may have contributed to the observed behavior, in line with previous reports describing the role of these components in modulating wine mouthfeel and polyphenol-related interactions in wine-like media (Brandão et al., 2017; Vidal, Francis, Williams, et al., 2004). Overall, differences in film thickness after HS addition should be interpreted at the individual sample level rather than as a uniform variety effect. While CS showed the highest film thickness, CS and CG did not differ clearly under the present conditions. CMR remained in a high-to-mid range, consistent with its classification in the present wine set as a non-drying/smooth reference and with previous reports associating Carménère with softer mouthfeel expression (Brossard et al., 2021).

**Table 2:** Average film thickness and friction coefficient of red wine samples and their mixtures with HS (1,1) across a speed range of 150 to 750 mm·s^−1^.

Sample	Film Thickness, FT (nm)			Coefficient of Friction, CoF (−)		
	Neat	With HS	*p*-value	Neat	With HS	*p*-value
CMR:ND	10.4 ± 2.8 ^abB^	27.5 ± 6.0 ^abA^	0.0021	0.23 ± 0.01 ^aA^	0.14 ± 0.03 ^bcB^	0.0018
CG:LD	6.5 ± 1.3 ^bcB^	26.2 ± 2.1 ^abA^	<0.0001	0.23 ± 0.01 ^aA^	0.16 ± 0.02 ^abcB^	0.0009
CG:HD	4.7 ± 0.7 ^cB^	20.1 ± 8.5 ^abA^	0.0109	0.16 ± 0.02 ^bB^	0.20 ± 0.02 ^abA^	0.0378
CS:LD	5.6 ± 1.0 ^cB^	16.5 ± 5.4 ^bA^	0.0070	0.16 ± 0.04 ^b^	0.12 ± 0.03 ^c^	0.1386
CS:MD	12.0 ± 3.2 ^aB^	34.4 ± 4.8 ^aA^	0.0003	0.11 ± 0.03 ^c^	0.16 ± 0.04 ^abcd^	0.0884
CS:HD	10.0 ± 0.2 ^abB^	28.0 ± 10.0 ^abA^	0.0114	0.11 ± 0.01 ^cB^	0.20 ± 0.00 ^aA^	<0.0001
*p*-value^a^	<0.0001	0.0195		<0.0001	0.0024	
W	10.1 ± 1.4			0.40 ± 0.02		
HS	25.0 ± 3.0			0.41 ± 0.01		

a Results are represented by the mean ± standard deviation of the film thickness and CoF obtained for the mean speed from 150 to 750 mm·s⁻¹, compared by ANOVA (*p*<0.05). Different lowercase letters in the same column indicate significant differences (*p*<0.05, Tukey). Different uppercase letters in a row indicate significant differences (*p*<0.05, Tukey). The nomenclature refers to the variety, CMR: Carménère, CG: Carignan; CS: Cabernet Sauvignon, and the astringency subquality of the drying type, ND: No Drying; LD: Low Drying; MD: Medium Drying; HD: High Drying. Controls (water and human saliva) were measured as neat lubricants under identical conditions and are reported for reference; no mixture and no *p*-values apply.

Regarding CoF, CG:LD decreased upon mixing with HS, whereas CG:HD increased. This reveals intra-varietal variability and suggests that the roughness/adhesiveness of aggregates — rather than their number alone — conditions friction in the mixed regime. For CS, only HD showed a significant increase in CoF after HS addition, whereas LD and MD did not differ significantly between neat wine and the corresponding wine–HS mixtures. In particular, HD shows the most significant increase with HS, consistent with aggregates that are less favorable to lubrication or have fewer continuous films. Across varieties, CMR markedly reduces CoF with HS (in line with relatively thick, stable films), whereas some CS and CG can increase CoF, reflecting distinct aggregation profiles.

Relative to the smooth/non-drying CMR baseline (FT↑/CoF↓), high-drying (HD) samples show thinner films and higher friction, consistent with salivary-film depletion and interfacial roughening. Nonetheless, a modulatory regime is evident: in some wines, soluble aggregates cooperate with saliva to sustain film integrity, so CoF does not necessarily increase and can even decrease despite substantial phenolic content (Pradal & Stokes, 2016; Sarkar & Krop, 2019). Within the stable 150–750 mm·s^−1^ range, friction is governed by the continuity of the lubricating film in the wine–HS mixture; therefore, more tannin does not necessarily mean higher CoF.

**Fig. 2:**
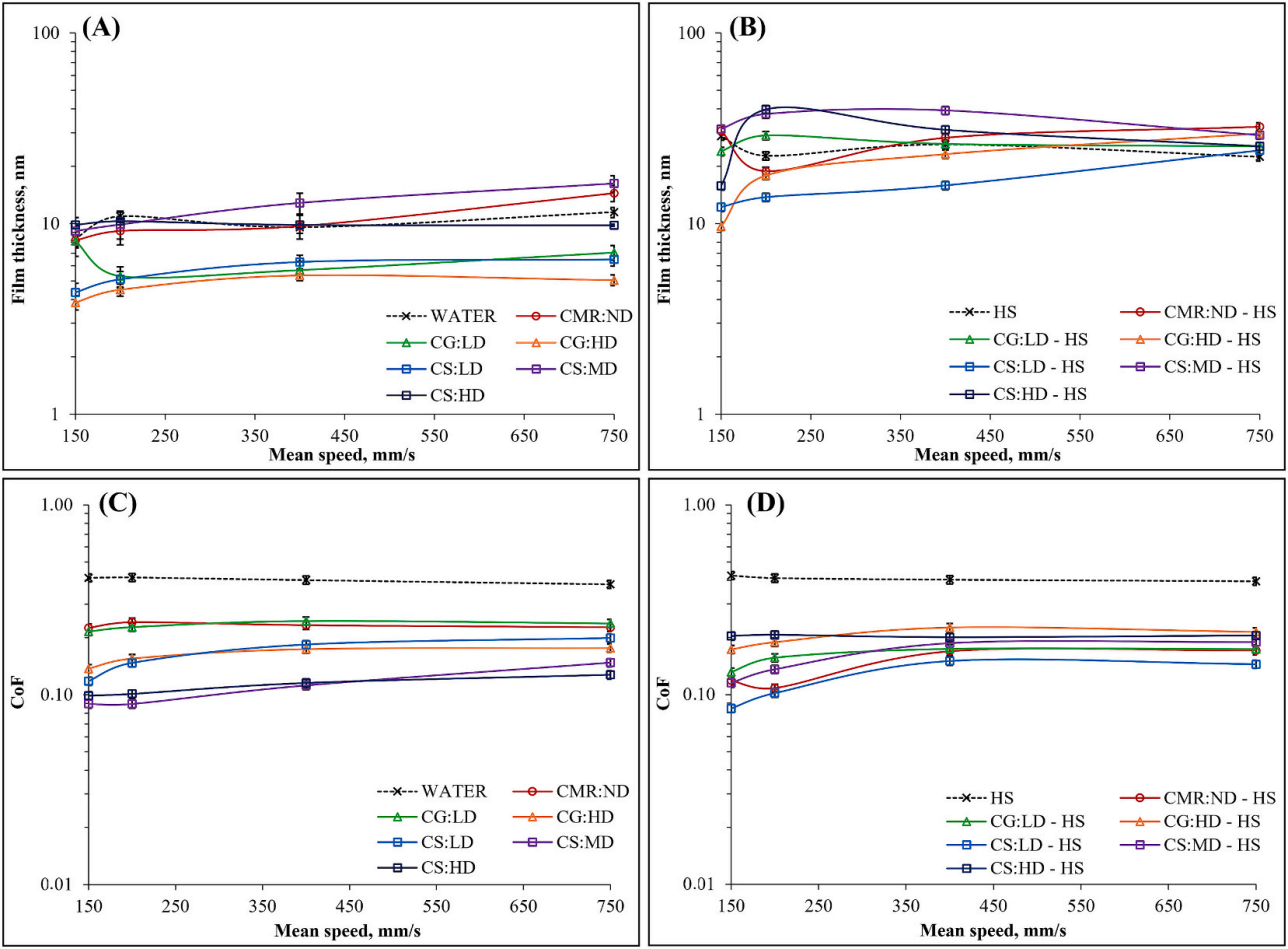
Comparison of film thickness and coefficient of friction: (A—C) between red wines and (B—D) between red wines – HS mixtures (1:1) from 150 to 750 mm·s^−1^. (For interpretation of the references to color in this figure legend, the reader is referred to the web version of this article.)

The speed-dependent behavior of the 1:1 wine–HS mixtures is shown in Fig. 2; the shaded 150–750 mm·s^−1^ window corresponds to a stable boundary→mixed regime in which the continuity of the entrained film primarily determines friction. Within this window, LD → HD progressions shift toward FT↓/CoF↑, whereas mixtures that retain a more stable and entrainable supernatant—characterized by a higher soluble fraction, smaller suspended colloids, and more negative ζ-potential—sustain higher FT and avoid large CoF rises. The concordance between FT and CoF supports a film-dominated mechanism rather than bulk-viscosity control alone (Sarkar et al., 2021; Stokes et al., 2011). We used a slightly higher normal load to stabilize the contact and suppress stick–slip that can arise at very low loads; this improves discrimination and repeatability without changing the qualitative ordering of wines within the 150–750 mm·s^−1^ window (Shewan et al., 2020).

Interfacial metrics after HS mixing (surface tension and contact angle on PDMS) are reported in Table 3 to assess wetting/spreading relevant to pellicle formation. Higher apparent contact angles on PDMS and/or higher surface tension indicate poorer wetting/spreading on this model surface, aligning with FT↓ and CoF↑; decreases in these metrics favor film formation and lower friction. Interfacial responses, therefore, provide a complementary route to discriminate drying levels and rationalize divergences among wines with similar tannin content (Stokes et al., 2013).

**Table 3:** Surface tension and contact angle of red wine and its mixtures with HS on a Polydimethylsiloxane (PDMS) surface.

Sample	SFT (mN/m)			Contact Angle (°)		
	Neat	With HS	*p*-value	Neat	With HS	*p*-value
CMR:ND	58.1 ± 0.6 ^aA^	49.5 ± 0.2 ^bB^	<0.0001	89.3 ± 2.5 ^c^	87.9 ± 4.3 ^c^	0.6505
CG:LD	53.0 ± 0.1 ^bA^	51.2 ± 0.1 ^aB^	<0.0001	105.0 ± 0.7 ^a^	102.7 ± 1.6 ^bc^	0.0832
CG:HD	49.2 ± 0.4 ^c^	49.3 ± 0.2 ^bcd^	0.6033	93.1 ± 3.7 ^bcB^	102.9 ± 0.6 ^bA^	0.0104
CS:LD	45.0 ± 0.2 ^dB^	48.9 ± 0.3 ^cA^	0.0001	99.8 ± 0.5 ^abB^	112.6 ± 1.0 ^aA^	<0.0001
CS:MD	52.7 ± 0.5 ^bA^	42.1 ± 0.1 ^dB^	<0.0001	102.1 ± 1.6 ^a^	102.2 ± 0.6 ^b^	0.9368
CS:HD	53.0 ± 0.7 ^bA^	49.4 ± 0.1 ^bcB^	0.0007	93.9 ± 3.5 ^bcB^	101.4 ± 0.4 ^bA^	0.0207
*p*-value^a^	<0.0001	<0.0001		<0.0001	<0.0001	
W	71.6 ± 0.3			117.0 ± 2.1		
HS	49.0 ± 0.2			109.6 ± 0.9		

a Results are represented by the mean ± standard deviation of the SFT and contact angle over a PDMS surface obtained by three repetitions, compared by ANOVA (*p*<0.05). Different lowercase letters in the same column indicate significant differences between red wines and between red wine – HS mixtures (1:1) at *p*<0.05 by Tukey's multiple comparisons. Different uppercase letters in the row indicate significant differences between red wine and the HS mixture (1:1) at p < 0.05 by Tukey's multiple comparisons. The nomenclature refers to the variety, CMR: Carménère, CG: Carignan; CS: Cabernet Sauvignon, and the astringency subquality of the drying type, ND: No Drying; LD: Low Drying; MD: Medium Drying; HD: High Drying. Controls (water and human saliva) were measured as neat lubricants under identical conditions and are reported for reference; no mixture and no *p*-values apply.

Among the CS wines, specifically LD and HD, the apparent contact angle on PDMS increases markedly after HS addition, indicating reduced wetting/spreading of the wine–HS mixtures on this model surface and potentially less favorable film replenishment under shear. CG shows differentiated responses: while HD increases the angle with HS, LD changes only marginally. These differences help explain the CoF behavior in the mixed regime. For CMR, contact angles remain more moderate and weakly sensitive to HS, consistent with a more stable film over the studied range.

Regarding SFT, the addition of HS can either raise or lower SFT, depending on the supernatant composition, thereby affecting the formation/stability of the lubricating film. For CS, opposite shifts are observed between samples (LD–HS ↑ whereas MD–HS ↓), suggesting supernatants with different compositions and distinct interfacial arrangements. CG and CMR tend to reduce SFT with HS, favoring film formation; however, the magnitude of this change does not always translate into lower CoF if the film structure is heterogeneous, in some cases, we observe bidirectional patterns, confirming that the interfacial response to HS is highly matrix-dependent for each wine.

Taken together, the physicochemical contrasts described in Table S-1 help contextualize the lubrication patterns observed in this section. Wines combining higher suspended turbidity, intermediate-to-high polysaccharide content, and moderate viscosity tended to preserve thicker films and lower friction after HS addition. In contrast, samples with weaker macromolecular support or less favorable wetting behavior shifted toward thinner films and higher CoF. In this sense, the present results indicate that drying is not governed solely by total phenolics, but by how the wine matrix supports an entrainable supernatant capable of spreading, replenishing the contact area, and maintaining film continuity under oral-inspired sliding conditions.

### 3.3 Microstructural characterization of wine–saliva aggregates

**Fig. 3:**
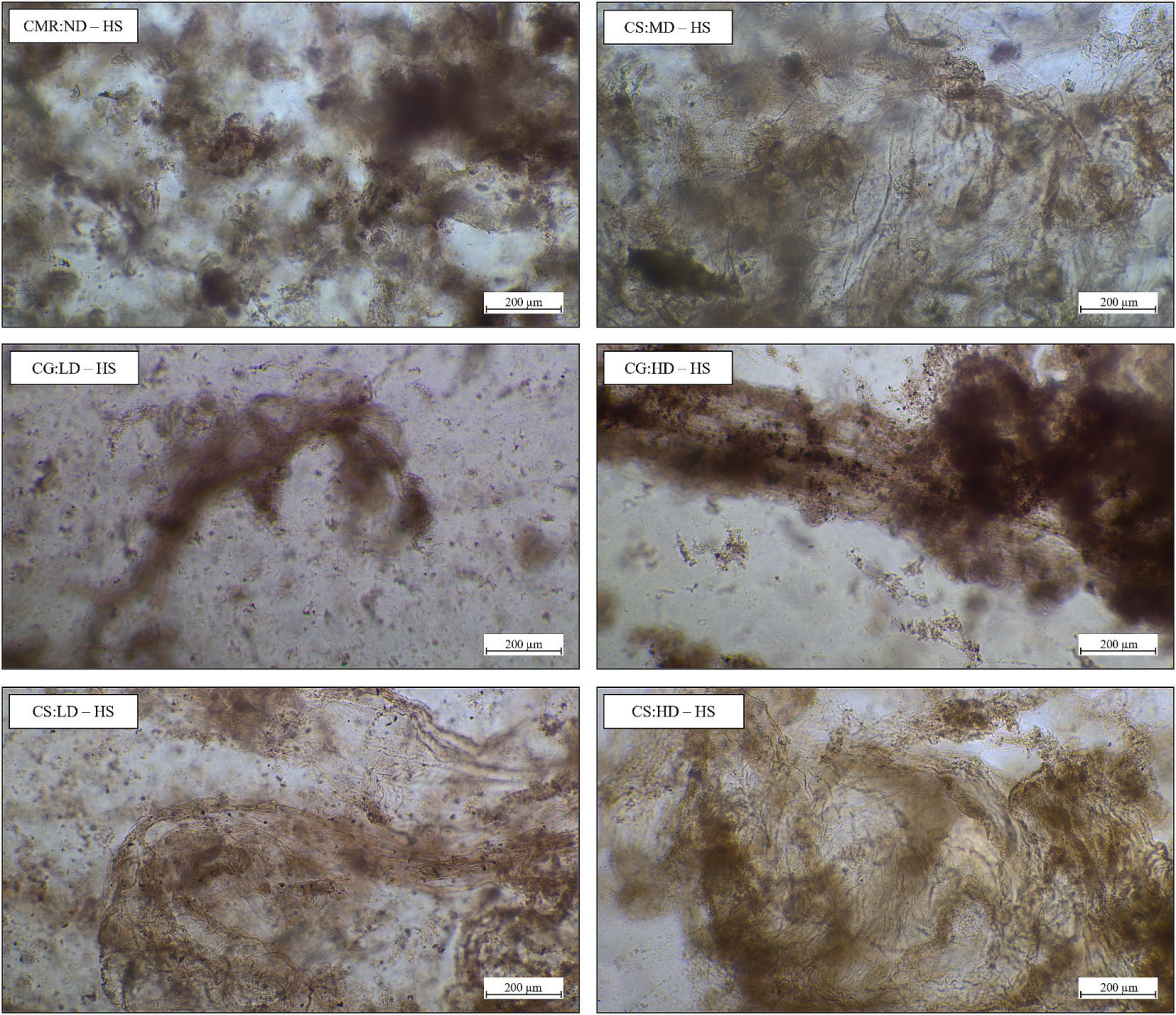
Light microscope micrographs of aggregates of wine – HS mixtures in a ratio 1:1. The nomenclature represents the mixture of HS with Carménère (CMR), Carignan (CG), and Cabernet Sauvignon (CS) with different intensity of the astringency subquality of the drying type; ND: No Drying; LD: Low Drying; MD: Medium Drying; HD: High Drying. The scale bar represents 200 μm.

Light micrographs of wine–HS aggregates (Fig. 3) reveal the morphology of the aggregates formed after mixing. We observe differences in size, compactness, and connectivity within and between varieties. For CG, LD displays finer, more dispersed aggregates than HD, consistent with the lower CoF measured in HS. In CS, HD tends to form larger, more connected aggregates than MD, which could hinder lubrication. CMR shows less dense structures, which aligns with its “non-drying” behavior and greater CoF reduction in HS. More generally, drying profiles tend to form larger, more irregular, and interconnected clusters, whereas smooth/non-drying profiles yield smaller, rounder, and more discrete entities. Beyond size alone, aggregate topology and connectivity are also important. Percolated networks bridge surface asperities and dissipate energy, making it harder to replenish the saliva pellicle; by contrast, compact colloids wet the surface more uniformly and are readily carried into the contact (Ployon et al., 2018; Pradal & Stokes, 2016). These microstructural signatures help explain differences in oral “volume” and the emergence of a smoother, less drying mouthfeel when smaller/rounder aggregates dominate the supernatant (Brossard et al., 2021).

Although no specific tannin-structure analyses were performed in the present study beyond general tannin quantification, the aggregate differences observed here are likely influenced not only by tannin concentration but also by tannin molecular architecture. Previous studies have shown that tannin–salivary protein interactions depend on structural features such as mean degree of polymerization (mDP), galloylation, B-ring hydroxylation, and stereochemistry, all of which may alter protein affinity and the balance between more soluble versus more precipitable complexes (González-Muñoz et al., 2021). In general, higher mDP and galloylation have been associated with stronger salivary protein binding, whereas increased B-ring trihydroxylation may reduce the effective interaction with salivary proteins. Matrix conditions may also contribute: lower pH tends to favor protein precipitation and astringency-related interactions, whereas higher ethanol can weaken tannin–protein precipitation and reduce overall astringency (Fontoin et al., 2008; González-Muñoz et al., 2021). Therefore, the differences in aggregate morphology and phase partitioning observed among wines may reflect the combined effects of unmeasured tannin structural features and matrix composition, in addition to total tannin content.

### 3.4 Role of aggregates and phase partitioning: supernatant vs pellet

The physicochemical composition of the supernatant (Table 4) is presented for the phase governing lubrication in the mixed regime. Three features track with film performance: (i) the fraction that remains soluble after HS mixing, (ii) colloidal stability—summarized by ζ-potential and hydrodynamic size (e.g., d_0.9_), and (iii) the macromolecular and phenolic payload (polysaccharides, proteins, TPI, tannins, anthocyanins). In CG wines, LD exhibits a more negative ζ-potential than HD, together with higher turbidity that remains in suspension. Although hydrodynamic size metrics did not indicate a clearly smaller colloidal size for LD than for HD, the more negative ζ-potential and the greater amount of suspended material suggest a more stable supernatant phase, consistent with thicker films and lower CoF, even though LD has higher wine tannins. By contrast, HD shows a less negative ζ-potential and lower suspended turbidity, indicating earlier destabilization and partial depletion of the supernatant. In Cabernet Sauvignon wines, CS:MD combined smaller particles, a more negative ζ-potential, and higher supernatant polysaccharide content, which favored more uniform film formation and a smaller increase in CoF. By contrast, HD presents larger sizes, less negative ζ, and lower suspended turbidity, consistent with supernatant depletion and higher CoF. CMR wines maintained intermediate ζ-potential and psize, a good soluble fraction, and elevated (but still suspended) turbidity, explaining their greater film thickness with HS and lower CoF, in line with their smooth/non-drying sensory role.

**Table 4:** Physicochemical characterization of supernatant (soluble aggregates) from the centrifugation of red wine – HS mixtures (1,1).

Sample	Supernatant				
	Supernatant	Hydrodynamic Diameter	d _0.9_	Zeta Potential, ζ	Total Phenols Index
	%	μm	μm	mV	OD 280 nm
CMR:ND	81.8 ± 0.7 ^ab^	7.1 ± 1.0 ^ab^	3.9 ± 1.3	−4.5 ± 0.9 ^a^	9.9 ± 0.4 ^bc^
CG:LD	75.7 ± 4.6 ^ab^	5.0 ± 0.2 ^abc^	2.7 ± 0.0	−8.4 ± 0.1 ^b^	11.8 ± 0.5 ^a^
CG:HD	75.1 ± 1.8 ^ab^	4.7 ± 0.4 ^bc^	2.7 ± 0.3	−2.0 ± 0.6 ^a^	10.0 ± 0.6 ^bc^
CS:LD	83.9 ± 1.0 ^a^	6.1 ± 0.4 ^abc^	3.3 ± 1.8	−3.3 ± 1.3 ^a^	9.0 ± 0.2 ^c^
CS:MD	73.2 ± 5.9 ^b^	3.3 ± 0.3 ^c^	3.1 ± 1.5	−8.3 ± 0.2 ^b^	11.1 ± 0.8 ^ab^
CS:HD	80.8 ± 2.7 ^ab^	8.2 ± 1.7 ^a^	5.6 ± 2.2	−1.9 ± 1.5 ^a^	12.2 ± 0.5 ^a^
*p*-value	0.0108	0.0095	0.3742	0.0010	<0.0001


Sample	Supernatant				
	Total Tannins	Anthocyanins Content	Turbidity	Polysaccharides Content	Protein Content
	g/L CE ^†^	mg/L	NTU	mg/L	mg/L
CMR:ND	0.15 ± 0.02 ^ab^	61 ± 1 ^bc^	28.0 ± 1.5 ^a^	131 ± 42	22 ± 1 ^a^
CG:LD	0.19 ± 0.05 ^a^	71 ± 1 ^a^	27.8 ± 4.8 ^a^	151 ± 33	23 ± 2 ^a^
CG:HD	0.10 ± 0.01 ^b^	62 ± 1 ^b^	10.6 ± 0.8 ^b^	179 ± 64	16 ± 1 ^b^
CS:LD	0.11 ± 0.01 ^b^	35 ± 2 ^e^	10.5 ± 0.7 ^b^	156 ± 10	11 ± 1 ^c^
CS:MD	0.15 ± 0.02 ^ab^	42 ± 2 ^d^	27.1 ± 0.7 ^a^	177 ± 19	17 ± 1 ^b^
CS:HD	0.12 ± 0.02 ^ab^	57 ± 2 ^c^	8.1 ± 0.7 ^b^	151 ± 27	11 ± 1 ^c^
*p*-value^a^	0.0123	<0.0001	<0.0001	0.6213	<0.0001

a Results are represented by the mean ± standard deviation of the supernatant physicochemical properties obtained by three repetitions compared by ANOVA (*p*<0.05). Different letters in the same column indicate significant differences between the mixtures at *p*<0.05 by Tukey's multiple comparisons. The nomenclature refers to the variety, CMR: Carménère, CG: Carignan; CS: Cabernet Sauvignon, and the astringency subquality of the drying type, ND: No Drying; LD: Low Drying; MD: Medium Drying; HD: High Drying. **^†^** Grams by liter catechin equivalent.

Low/non-drying profiles retain more phenolics and macromolecules in solution, exhibit more negative ζ potentials, and form smaller/rounder colloids, resulting in suspended turbidity. These conditions stabilize the film entering the contact (FT↑/CoF↓). Drying profiles display supernatant depletion (turbidity increases, then sediments, size increases, and |ζ| decreases), transferring interaction mass to the pellet and impairing lubrication. Thus, supernatant chemistry — not total tannins alone — predicts tribology (Brossard et al., 2021; Watrelot et al., 2017; Watrelot & Norton, 2020). Collectively, Table 4 substantiates a supernatant-centric route to smoother, less drying mouthfeel: stabilized, well-wetting colloids enable thicker films and lower friction across varieties.

The precipitated fraction (Table S-3) provides a diagnostic counterpoint. We report the insoluble fraction (%) and the composition of the pellet—TPI (total polyphenol index), tannins, anthocyanins, polysaccharides, and proteins. For Carignan (CG), LD shows higher TPI/tannins in the pellet than HD, yet its CoF with HS is lower; this indicates that “more pellet” does not necessarily mean higher friction in the mixed regime. In Cabernet Sauvignon (CS), MD and HD display high protein content in the pellet; however, their tribological impacts differ, suggesting that the pellet's quality/microstructure—not only its quantity—drives the outcome. Carménère (CMR) exhibits intermediate insoluble proportions, consistent with a more favorable tribological response. In general, heavier or denser pellets indicate greater depletion of the supernatant and tend to correlate with thinner films and higher friction; however, because the pellet is not entrained during sliding, its influence is indirect, representing how much colloidal mass has been removed from the lubricating phase (Brossard, Bordeu, Ibáñez, Chen and Osorio, 2020, Brossard et al., 2021; Prinz & Lucas, 2000).

### 3.5 Rheological behavior of the aggregates

Ostwald–de Waele fits (*K*,n) and RTA for supernatant and pellet are provided in Table S-4, and G′/G″ and apparent viscosities are detailed in Figs. S-1 and S-2. Supernatant (all varieties) shows a low K with n ≈ 0.2–0.7, indicating shear-thinning complex fluids. Lower K + higher n are associated with lower CoF in the mixed regime because more fluid, yet continuous films are entrained. Heterogeneous RTA points to distinct flow memory across wines (matrix-dependent thixotropy). Precipitates (all varieties) exhibit a very high K and low n (≈0.04–0.37), indicating cohesive particulate networks. Elevated RTA suggests hysteresis that could increase friction under boundary conditions. Differences between CG and CS point to distinct aggregate architectures. G′ > G″ in some HD pellets indicates more solid-like behavior, whereas G″ dominates in supernatants, consistent with liquid-like lubricating phases. The CMR supernatant pairs low K with mid-to-high n and very low RTA, a triad that favors entrainment and film continuity, matching FT↑/CoF↓. Its pellet shows high K (as expected for cohesive networks), but modest RTA compared with certain CS/CG pellets, consistent with a less disruptive boundary contribution. This rheological signature explains why CMR behaves as a non-drying anchor despite intermediate phenolic levels: the entrainable phase is rheologically ‘forgiving’ (Brandão et al., 2017; Brossard et al., 2020). Therefore, supernatant rheology—rather than pellet strength—aligns with lower friction, reinforcing the film-dominated mechanism in Table 3 (contact angle/SFT) (Sarkar et al., 2021; Sarkar & Krop, 2019). Because rheology was measured in duplicate, these results are used primarily to contextualize tribological trends and are interpreted with caution, especially in multivariate summaries.

Steady-shear apparent viscosity (η) at 1, 50, and 100 s^−1^ is summarized in Fig. S-2 (with corresponding parameters in Table S-5) for the supernatant (entrainable phase) and, where relevant, the pellet. The selected rates capture: 1 s^−1^ (low-shear structure and weak network persistence), 50 s^−1^ (oral-like shear during tongue–palate motions), and 100 s^−1^ (upper bound for rapid bolus movement) (Badia-Olmos et al., 2022; Cichero et al., 2017). Across matrices, curves display shear-thinning, consistent with the flow indices (*n* < 1) in Table S-4 and the thixotropy inferred from RTA. This pattern aligns with the oral processing literature, which shows that moderate shear promotes entrainment and reduces dissipation within the lubricating film (Shewan et al., 2020; Stokes et al., 2013). At 50 s^−1^, where film formation is most predictive of tribological outcomes, lower-to-moderate η in the supernatant typically coincides with higher FT and lower CoF, provided the interfacial conditions (contact angle/SFT) support wetting. A very high η can hinder spreading and replenishment, while a too low η can yield films that are easily depleted under load; the favorable window thus reflects a balance between the fluidity and cohesion of the entrained phase (Stokes et al., 2013). This explains why viscosity alone does not determine friction; it must be interpreted in conjunction with ζ-potential, size, and interfacial metrics (Table 3).

For CG at 50 s^−1^, LD tends to exhibit lower-to-moderate η values in the supernatant than HD, consistent with its thicker films and lower CoF than HS. The small-to-moderate η at oral shear suggests readily entrained colloids that maintain continuity without over-damping. CS:MD often shows the lowest η₅₀ among CS supernatants, consistent with the greatest FT observed for CS–HS and with more negative ζ and smaller sizes (Table 4). HD tends to retain a higher η at 1–50 s^−1^ but without the interfacial advantages (less negative ζ, larger size), leading to higher CoF despite viscosity alone not being extreme. The CMR supernatant exhibits intermediate η₁ and modest η₅₀–η₁₀₀ values with tight dispersion, a signature that favors entrainment and stable spreading. Together with its intermediate-to-negative ζ value and moderate size (Table 4), this rheological profile is consistent with the FT↑/CoF↓ ranking and its role as the non-drying reference.

Pellet η at 1–100 s^−1^ is frequently high, reflecting cohesive particulate networks (Table S-5). However, because the pellet is not entrained in the contact during sliding, its viscosity primarily indicates the amount of mass remaining in the supernatant. Accordingly, pellets with very high η and large RTA corroborate supernatant depletion and help explain boundary-like friction increases in drying profiles—indirectly, by reducing what is available to lubricate (Brossard et al., 2020). At oral-like shear (≈50 s^−1^), supernatant apparent viscosity supports film entrainment when paired with favorable ζ-potential/size and interfacial wetting; this triad aligns with FT↑/CoF↓, particularly for CMR and CS–MD, whereas CG–HD/CS–HD deviate due to supernatant depletion and/or less favorable interfacial conditions.

### 3.6 Multivariate analysis (PCA)

**Fig. 4:**
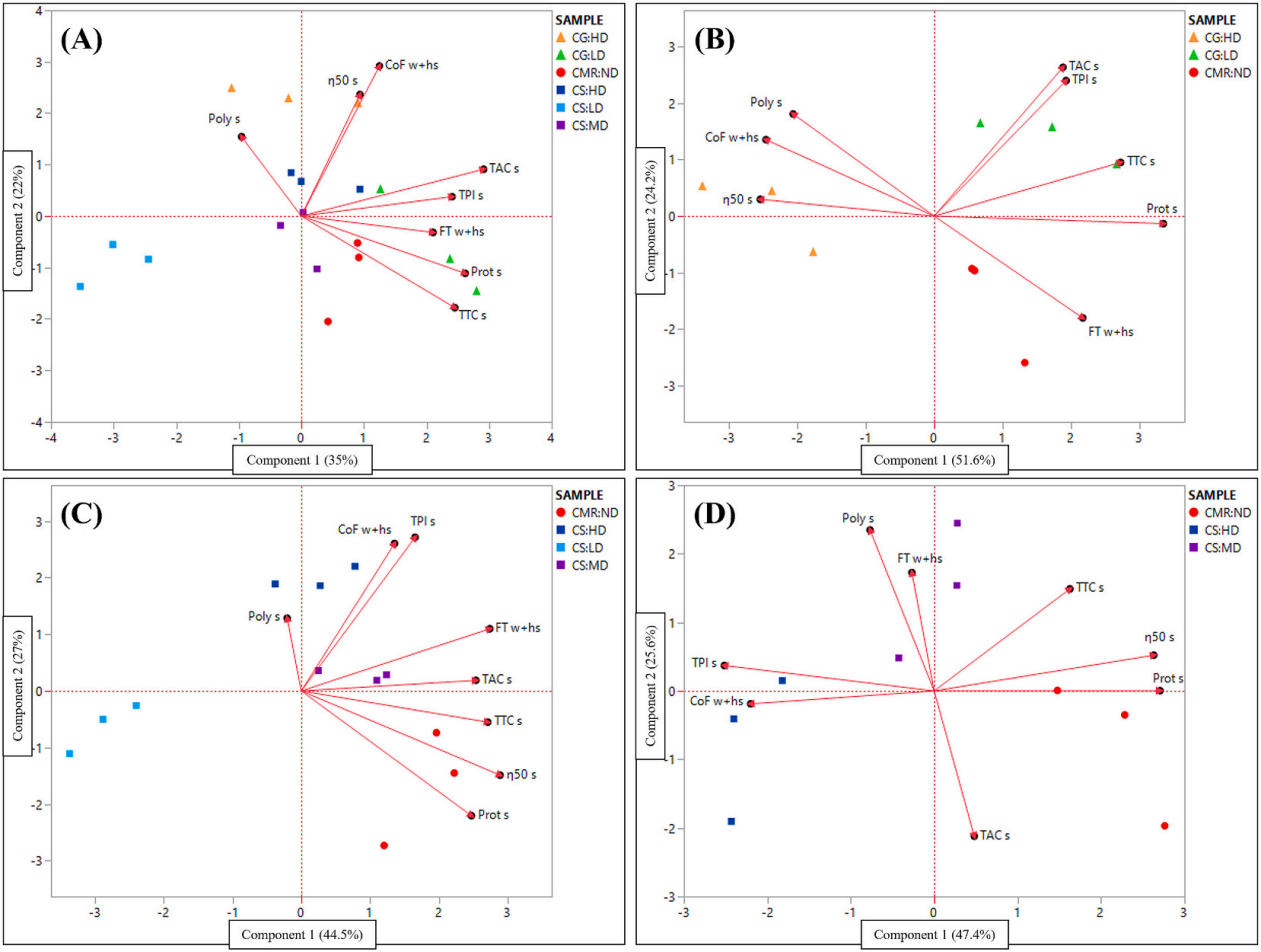
PCA of the red wines: (A) Carménère-ND, Carignan-LD-HD and Cabernet Sauvignon-LD-MD-HD, (B) Carménère-ND and Carignan-LD-HD, (C) Carménère-ND and Cabernet Sauvignon -LD-MD-HD (D) Carménère -ND and Cabernet Sauvignon -MD -HD, including the physical properties from the mixture with HS (CoF_w+hs_; FT_w+hs_; η_50 supernatant_), and chemical properties (Poly _supernatant_; Prot _supernatant_; TPI _supernatant_; TTC _supernatant_; TAC _supernatant_). (For interpretation of the references to color in this figure legend, the reader is referred to the web version of this article.)

We used PCA (Fig. 4) as an exploratory multivariate visualization to relate wine composition to the physicochemical properties of the supernatants and to illustrate how these properties co-vary with mixture-level lubrication (FTw + hs, CoFw+hs) in the 150–750 mm·s^−1^ window. Given the limited number of wines (*n* = 6), PCA is interpreted here as an illustrative tool for pattern recognition rather than as a basis for strong inferential claims. Variables were autoscaled, and collinearity was screened; tribology (FTw + hs, CoFw+hs) was measured on 1:1 wine–HS mixtures, whereas polysaccharides, proteins, TPI, TTC, TAC, and, where indicated, supernatant η₅₀ were measured after mixing and phase separation. Replicate points were retained in the score plots only to visualize the experimental dispersion associated with each wine across the analytical workflow; they were not intended to increase the effective number of wines or to support independent inferential testing. Rheological variables (e.g., η₅₀) are included for exploratory visualization and should be interpreted with caution due to duplicate measurements. This multivariate approach remains consistent with the established link between oral shear/tribology and mouthfeel, as well as with the supernatant-centric view of wine–saliva aggregation (Brossard et al., 2021; Laguna et al., 2019; Sarkar & Krop, 2019; Stokes et al., 2013; Watrelot & Norton, 2020). Carménère (CMR, non-drying reference) was retained as an anchor in all panels. This interpretation is consistent with the PCA, where wines with higher polysaccharide support, moderate viscosity, and more favorable supernatant properties clustered toward the lower-friction / higher-film-thickness side, whereas samples with less favorable colloidal balance shifted toward the friction-dominated region.

In Panel A (all wines), PC1 appears to capture a film-continuity vs. friction contrast, with FT_w+hs_ loading in the opposite direction to CoF_w+hs_, consistent with a film-dominated mechanism in which wettability and entrainment govern mixed-regime friction (Pradal & Stokes, 2016; Stokes et al., 2011). PC2 tends to contrast matrices with greater supernatant macromolecular support and moderate η₅₀ (favoring spreading/replenishment at oral-like shear) against phenolic-dominated patterns that do not, by themselves, guarantee good lubrication (Badia-Olmos et al., 2022; Brandão et al., 2017; Cichero et al., 2017; Sarkar et al., 2021; Vidal, Francis, Noble, et al., 2004; Vidal, Francis, Williams, et al., 2004). In this map, CMR is located in the low-friction/high-film sector; CS-MD typically projects nearby, CS-LD is intermediate, and CS-HD (with some CG-HD) tends to fall toward the CoF-dominated side, where FT is reduced—consistent with sensory/chemical associations reported for red wines (Pavéz et al., 2022) and with a supernatant-centered interpretation (Brossard et al., 2021; Watrelot & Norton, 2020).

In Panel B (CMR + CG), the within-variety difference is more apparent: CG-LD tends to plot toward higher FT_w+hs_ / lower CoF_w+hs_ and aligns with more negative zeta, smaller supernatant size, and stronger macromolecular support, whereas CG-HD tends to fall toward the friction-dominated side. This pattern is consistent with the idea that, even at similar or higher wine-level phenolics, the state of the soluble fraction after HS mixing contributes to differentiating less-drying from more-drying Carignan (Brossard et al., 2021; Watrelot & Norton, 2020).

In Panel C (CMR + CS, LD–MD–HD), a LD → MD → HD trend is suggested when using a fixed non-drying anchor. CS-MD tends to plot near CMR, pairing higher FT_w+hs_ and lower CoF_w+hs_ with more supernatant polysaccharides/proteins and moderate η₅₀ features consistent with stable, wetting colloids in the entrained phase (Brandão et al., 2017; Sarkar et al., 2021; Watrelot et al., 2017). CS-LD is intermediate, while CS-HD tends to shift toward CoF(+) / FT(−) with larger size, less negative zeta, and lower suspended turbidity, consistent with supernatant depletion despite substantial phenolic potential; the relative positioning remains compatible with mixed-regime tribology governed by film coverage rather than bulk viscosity alone (Pradal & Stokes, 2016; Stokes et al., 2011).

In Panel D (CMR + CS, MD vs HD), isolating CS-MD and CS-HD against CMR retains the PC1 separation (FT_w+hs_↑ opposite CoF_w+hs_↑) and highlights the potential role of supernatant stability and the flow window (η₅₀) in whether the mixture builds a continuous, replenishable film (Badia-Olmos et al., 2022; Cichero et al., 2017; Sarkar et al., 2021). This subset broadly mirrors the global layout and preserves the qualitative ordering of samples, consistent with earlier reports linking chemistry, colloids, and perceived astringency (Brossard et al., 2021; Pavéz et al., 2022).

Overall, the four panels provide an illustrative summary: wines whose supernatants remain well-stabilized and moderately viscous at oral-like shear tend to associate with higher FT_w+hs_ and lower CoF_w+hs_ (CMR, CS-MD, CG-LD), whereas samples showing supernatant depletion or sub-optimal wetting/size–charge balance tend to fall toward the friction-dominated side (CS-HD, some CG-HD). The PCA offers a compact visualization of the proposed chemistry → supernatant → lubrication pathway with CMR as a stable non-drying reference (Brossard et al., 2021; Pradal & Stokes, 2016; Stokes et al., 2011; Watrelot & Norton, 2020). Interpretation is qualitative, and the main mechanistic conclusions are supported primarily by the univariate results above. For visualization of the correlation behavior between the precipitate parameters, see Fig. S-3.

## 4 Conclusions

Across sections, the evidence converges on a supernatant-centric control of drying: composition sets aggregation propensity, but perception depends on (i) how much interaction mass remains soluble, (ii) its interfacial behavior (wetting/contact angle and zeta potential), and (iii) the amount and morphology of saliva–polyphenol aggregates that populate the lubricating gap. In line with the mechanism proposed by our working group, higher aggregate abundance may contribute to perceived intensity, whereas aggregate form/surface features can help define the perceived subquality. This framework reconciles cases in which high tannin does not yield strong drying because favorable supernatant colloids preserve the entrainable lubricating phase. From a design perspective, steering the macromolecular balance — particularly the polysaccharide pool — toward smaller/rounder colloids with more negative zeta potential may shift mouthfeel toward ‘velvet’ without necessarily lowering phenolic content.

Methodologically, chemically inert and robust tribopairs, together with a higher normal load, improved repeatability and sharpened discrimination without altering the qualitative ranking of wines, lending confidence to the mechanistic interpretation. Future work should increase biomimicry by employing mucin-coated compliant elastomers (e.g., PDMS) or hydrogels with oral-like roughness and elasticity, while controlling temperature (∼28 °C), pH, and ionic strength, and operating under physiologically relevant loads, speeds, and saliva replenishment. Coupling FT/CoF with in situ film-thickness interferometry or fluorescence, together with high-speed imaging, would help resolve how aggregate topology and pellicle renewal co-determine friction. Finally, targeted profiling of polysaccharide families (AGPs, RG-I/II, mannoproteins) is recommended to identify the macromolecules that most effectively stabilize the lubricating phase. As a first step, the present study focused on a limited set of wines spanning drying intensity; extending the approach to more varieties, vintages, and winemaking conditions, and to additional astringency subqualities (e.g., grippy, rough, adhesive), will be essential to generalize the proposed mechanism and strengthen predictive capability across wine styles.

## CRediT authorship contribution statement

**Emerson Núñez:** Writing – original draft, Software, Investigation, Formal analysis, Data curation. **Edmundo Bordeu:** Writing – review & editing, Validation, Supervision, Conceptualization. **Max Marian:** Validation, Software, Methodology. **Martin Vrbka:** Writing – review & editing, Resources. **David Nečas:** Validation, Software, Methodology. **Fernando Osorio:** Supervision, Methodology, Investigation. **Andreas Rosenkranz:** Validation, Supervision, Methodology. **V. Felipe Laurie:** Visualization, Supervision, Investigation. **Natalia Brossard:** Writing – review & editing, Supervision, Project administration, Funding acquisition, Conceptualization.

## Funding

This work was funded by VitiScience – CIA 250013 and FOVI 230072, supported by the National Agency for 10.13039/100006190Research and Development (ANID), Chile.

## Declaration of competing interest

The authors declare that they have no known competing financial interests or personal relationships that could have appeared to influence the work reported in this paper.

## Data Availability

Data will be made available on request.
