# Supplementary material for: Interfacial and colloidal drivers of wine drying astringency: the role of wine–saliva aggregates
Source: Food Chem X. 2026 Jul 26;38:104223. doi: 10.1016/j.fochx.2026.104223 (PMC13452222; doi:10.1016/j.fochx.2026.104223)
Supplement: Supplementary file 1 [file mmc1.docx]

**Table S-1.** Physicochemical composition of red wines with different astringency intensity based on drying subquality.

| **Wine** | **Turbidity** | **Total Phenols Index** | **Total Tannins** | **Anthocyanins** | **Polysaccharides Content** | **Protein Content** | **Viscosity, ×10^3^** |
| --- | --- | --- | --- | --- | --- | --- | --- |
|  | **NTU** | **OD 280 nm** | **g/L CE ^†^** | **mg/L** | **mg/L** | **mg/L** | **Pa s** |
| CMR:ND | 2.6 ± 0.2 ^c^ | 35.3 ± 0.6 ^de^ | 0.44 ± 0.02 ^e^ | 231 ± 9 ^b^ | 229 ± 26 ^ab^ | 43 ± 11 | 1.60 ± 0.03 ^ab^ |
| CG:LD | 8.1 ± 0.3 ^a^ | 55.5 ± 1.7 ^a^ | 1.08 ± 0.03 ^a^ | 320 ± 9 ^a^ | 215 ± 45 ^b^ | 36 ± 9 | 1.59 ± 0.01 ^ab^ |
| CG:HD | 8.0 ± 0.3 ^a^ | 41.1 ± 0.6 ^cd^ | 0.62 ± 0.04 ^c^ | 221 ± 9 ^bc^ | 234 ± 86 ^ab^ | 42 ± 8 | 1.65 ± 0.01 ^a^ |
| CS:LD | 2.9 ± 0.4 ^c^ | 34.0 ± 0.2 ^e^ | 0.53 ± 0.02 ^d^ | 140 ± 10 ^d^ | 267 ± 41 ^ab^ | 43 ± 1 | 1.30 ± 0.06 ^c^ |
| CS:MD | 2.7 ± 0.2 ^c^ | 47.7 ± 4.3 ^b^ | 0.71 ± 0.04 ^b^ | 217 ± 7 ^bc^ | 394 ± 82 ^a^ | 40 ± 2 | 1.60 ± 0.11 ^ab^ |
| CS:HD | 5.8 ± 0.2 ^b^ | 44.7 ± 2.6 ^bc^ | 0.64 ± 0.03 ^bc^ | 209 ± 4 ^c^ | 358 ± 58 ^ab^ | 44 ± 5 | 1.59 ± 0.04 ^bc^ |
| **p-value** | **<0.0001** | **<0.0001** | **<0.0001** | **<0.0001** | **0.0138** | 0.7465 | **0.0039** |

Results are represented by the mean ± standard deviation of the physicochemical composition of red wines, compared by ANOVA (p<0.05). Different lowercase letters in the same column indicate significant differences between red wines at p<0.05 by Tukey's multiple comparisons. The nomenclature refers to the variety, CMR: Carménère, CG: Carignan; CS: Cabernet Sauvignon, and the astringency sub-quality of the drying type, ND: No Drying; LD: Low Drying; MD: Medium Drying; HD: High Drying. **^†^** Grams by liter catechin equivalent.

**Table S-2.** Physicochemical composition of red wines with different astringency intensity based on drying subquality (*Continuation*).

| **Wine** | **Alcohol** | **Titratable Acidity** | **Volatile**  **Acidity** | **pH** | **Residual Sugars** | **Color Intensity** | **Hue** | **Total Sulfur Dioxide** | **Free Sulfur Dioxide** |
| --- | --- | --- | --- | --- | --- | --- | --- | --- | --- |
|  | **% v/v** | **g/L _H2SO4_** | **g/L _CH3COOH_** |  | **g/L Glucose** |  |  | **mg/L** | **mg/L** |
| CMR:ND | 13.2 ± 0.1 ^d^ | 3.84 ± 0.02 ^c^ | 0.29 ± 0.02 ^d^ | 3.48 ± 0.01 ^b^ | 1.86 ± 0.02 ^d^ | 11.1 ± 0.2 ^e^ | 0.45 ± 0.00 ^c^ | 39.7 ± 1.3 ^a^ | 14.5 ± 1.6 |
| CG:LD | 14.4 ± 0.1 ^a^ | 4.71 ± 0.02 ^a^ | 0.44 ± 0.02 ^b^ | 3.30 ± 0.01 ^d^ | 2.38 ± 0.02 ^b^ | 25.2 ± 0.4 ^a^ | 0.47 ± 0.02 ^c^ | 38.4 ± 1.3 ^ab^ | 18.4 ± 1.5 |
| CG:HD | 14.3 ± 0.1 ^b^ | 4.16 ± 0.04 ^b^ | 0.37 ± 0.01 ^c^ | 3.30 ± 0.01 ^d^ | 2.37 ± 0.01 ^b^ | 15.7 ± 0.1 ^c^ | 0.50 ± 0.02 ^bc^ | 38.0 ± 2.0 ^ab^ | 17.0 ± 2.0 |
| CS:LD | 13.0 ± 0.1 ^e^ | 3.54 ± 0.01 ^d^ | 0.39 ± 0.02 ^c^ | 3.50 ± 0.01 ^ab^ | 2.04 ± 0.01 ^c^ | 13.4 ± 0.2 ^d^ | 0.55 ± 0.03 ^b^ | 36.5 ± 1.3 ^ab^ | 14.7 ± 0.6 |
| CS:MD | 14.4 ± 0.1 ^ab^ | 3.88 ± 0.02 ^c^ | 0.35 ± 0.02 ^c^ | 3.42 ± 0.01 ^c^ | 2.06 ± 0.02 ^c^ | 16.8 ± 0.1 ^b^ | 0.34 ± 0.00 ^d^ | 34.8 ± 1.3 ^b^ | 17.3 ± 1.7 |
| CS:HD | 14.1 ± 0.1 ^c^ | 3.22 ± 0.02 ^e^ | 0.53 ± 0.01 ^a^ | 3.51 ± 0.02 ^a^ | 3.03 ± 0.02 ^a^ | 6.6 ± 0.2 ^f^ | 0.64 ± 0.04 ^a^ | 40.5 ± 1.9 ^a^ | 16.4 ± 1.3 |
| **p-value** | **<0.0001** | **<0.0001** | **<0.0001** | **<0.0001** | **<0.0001** | **<0.0001** | **<0.0001** | **0.0063** | 0.00547 |

Results are represented by the mean ± standard deviation of the physicochemical composition of red wines, compared by ANOVA (p<0.05). Different lowercase letters in the same column indicate significant differences between red wines at p<0.05 by Tukey's multiple comparisons. The nomenclature refers to the variety, CMR: Carménère, CG: Carignan; CS: Cabernet Sauvignon, and the astringency sub-quality of the drying type, ND: No Drying; LD: Low Drying; MD: Medium Drying; HD: High Drying.

**Table S-3.** Chemical characterization of precipitate (insoluble aggregates) from the centrifugation of red wine – human saliva mixtures (1:1).

| **Sample** | **Precipitate** | | | | | |
| --- | --- | --- | --- | --- | --- | --- |
|  | **Precipitate** | **Total Phenols Index** | **Total Tannins** | **Anthocyanins Content** | **Polysaccharides Content** | **Protein Content** |
|  | **%** | **OD 280 nm** | **g/L CE ^†^** | **mg/L** | **mg/L** | **mg/L** |
| CMR:ND | 18.2 ± 0.7 ^ab^ | 25.4 ± 0.8 ^c^ | 0.29 ± 0.04 ^c^ | 170 ± 9 ^bc^ | 99 ± 43 ^b^ | 21 ± 12 |
| CG:LD | 24.3 ± 4.6 ^ab^ | 43.7 ± 2.0 ^a^ | 0.89 ± 0.07 ^a^ | 249 ± 10 ^a^ | 64 ± 46 ^b^ | 13 ± 11 |
| CG:HD | 24.9 ± 1.8 ^ab^ | 31.1 ± 1.1 ^bc^ | 0.52 ± 0.03 ^bc^ | 159 ± 9 ^bc^ | 55 ± 53 ^b^ | 26 ± 8 |
| CS:LD | 16.1 ± 1.0 ^b^ | 25.1 ± 0.4 ^c^ | 0.43 ± 0.01 ^c^ | 106 ± 10 ^d^ | 111 ± 51 ^ab^ | 31 ± 1 |
| CS:MD | 26.8 ± 5.9 ^a^ | 36.6 ± 5.1 ^b^ | 0.57 ± 0.03 ^b^ | 176 ± 6 ^b^ | 217 ± 99 ^a^ | 24 ± 3 |
| CS:HD | 19.2 ± 2.7 ^ab^ | 32.5 ± 2.7 ^b^ | 0.52 ± 0.02 ^bc^ | 152 ± 4 ^c^ | 207 ± 55 ^a^ | 33 ± 5 |
| **p-value** | **0.0108** | **<0.0001** | **<0.0001** | **<0.0001** | **0.0222** | 0.0857 |

Results are represented by the mean ± standard deviation of the Precipitate (insoluble aggregates) physicochemical properties obtained by three repetitions compared by ANOVA (p<0.05). Different letters in the same column indicate significant differences between the mixtures at p<0.05 by Tukey's multiple comparisons. The nomenclature refers to the variety, CMR: Carménère, CG: Carignan; CS: Cabernet Sauvignon, and the astringency sub-quality of the drying type, ND: No Drying; LD: Low Drying; MD: Medium Drying; HD: High Drying. **^†^** Grams by liter catechin equivalent.

**Table S-4.** Parameters of the Ostwald de Waele model fitted to the shear viscosity data supernatant (R^2^ 0.8 – 0.98) and precipitate (R^2^ 0.93 – 0.99).

| **Ostwald de Waele fit: η(*y*) = K(y)^n-1^** | | | | | | | | |
| --- | --- | --- | --- | --- | --- | --- | --- | --- |
| **Sample** | **K (Pa.s^n^)** | |  | **n (-)** | |  | **Relative Thixotropic Area RTA (%)** | |
|  | **Supernatant** | **Precipitate** |  | **Supernatant** | **Precipitate** |  | **Supernatant** | **Precipitate** |
| CMR:ND | 0.02 ± 0.01 ^c^ | 25.96 ± 7.73 ^ab^ |  | 0.53 ± 0.08 ^ab^ | 0.07 ± 0.01 ^d^ |  | 2.2 ± 0.6 ^b^ | 5.1 ± 3.0 ^cd^ |
| CG:LD | 0.05 ± 0.01 ^b^ | 39.48 ± 15.59 ^a^ |  | 0.29 ± 0.03 ^ab^ | 0.04 ± 0.00 ^d^ |  | 0.8 ± 0.1 ^b^ | 15.7 ± 0.4 ^b^ |
| CG:HD | 0.13 ± 0.01 ^a^ | 7.09 ± 1.38 ^b^ |  | 0.20 ± 0.09 ^b^ | 0.16 ± 0.01 ^c^ |  | 19.0 ± 5.5 ^a^ | 26.4 ± 0.9 ^a^ |
| CS:LD | 0.01 ± 0.00 ^c^ | 47.28 ± 4.82 ^a^ |  | 0.63 ± 0.04 ^ab^ | 0.27 ± 0.02 ^b^ |  | 22.0 ± 0.4 ^a^ | 11.1 ± 1.5 ^bc^ |
| CS:MD | 0.02 ± 0.01 ^c^ | 23.65 ± 1.37 ^ab^ |  | 0.60 ± 0.21 ^ab^ | 0.17 ± 0.03 ^c^ |  | 3.6 ± 1.8^b^ | 1.5 ± 0.0 ^d^ |
| CS:HD | 0.01 ± 0.00 ^c^ | 23.95 ± 3.37 ^ab^ |  | 0.70 ± 0.28 ^a^ | 0.37 ± 0.02 ^a^ |  | 19.2 ± 0.9 ^a^ | 17.4 ± 1.9 ^b^ |
| **p-value** | **<0.0001** | **0.0179** |  | **0.0394** | **<0.0001** |  | **0.0003** | **<0.0001** |
| HS | 0.03 ± 0.00 | |  | 0.38 ± 0.02 | |  | 2.3 ± 0.1 | |

Results are represented by the mean ± standard deviation of the Supernatant and Precipitate Ostwald de Waele fit (K, n, RTA) obtained by three repetitions compared by ANOVA (p<0.05). Different letters in the same column indicate significant differences between the samples at p<0.05 by Tukey's multiple comparisons. The nomenclature refers to the variety, CMR: Carménère, CG: Carignan; CS: Cabernet Sauvignon, and the astringency sub-quality of the drying type, ND: No Drying; LD: Low Drying; MD: Medium Drying; HD: High Drying. Flow behavior curves of shear stress as a function of shear rate fitted to the power law model.

**Table S-5.** Apparent viscosity (η) of red wine with human saliva (1:1) separated by centrifugation in supernatant (soluble aggregates) and precipitate (insoluble aggregates) at shear rates of 1 s^−1^, 50 s^−1^, and 100 s^−1^.

| **Sample** |  | **Supernatant (×10^2^)** | | |  | **Precipitate** | | |
| --- | --- | --- | --- | --- | --- | --- | --- | --- |
|  |  | **η_1_ (Pa·s)** | **η_50_ (Pa·s)** | **η_100_ (Pa·s)** |  | **η_1_ (Pa·s)** | **η_50_ (Pa·s)** | **η_100_ (Pa·s)** |
| CMR:ND |  | 1.5 ± 0.0 ^c^ | 0.2 ± 0.0 ^ab^ | 0.2 ± 0.0 |  | 26.0 ± 7.7 ^ab^ | 0.7 ± 0.2 ^c^ | 0.4 ± 0.1 ^b^ |
| CG:LD |  | 4.5 ± 0.0 ^b^ | 0.3 ± 0.0 ^ab^ | 0.2 ± 0.0 |  | 39.5 ± 15.6 ^a^ | 0.9 ± 0.4 ^bc^ | 0.5 ± 0.2 ^b^ |
| CG:HD |  | 12.3 ± 0.0 ^a^ | 0.5 ± 0.0 ^a^ | 0.3 ± 0.0 |  | 7.1 ± 1.4 ^c^ | 0.3 ± 0.0 ^c^ | 0.1 ± 0.0 ^b^ |
| CS:LD |  | 0.6 ± 0.0 ^c^ | 0.1 ± 0.0 ^b^ | 0.1 ± 0.0 |  | 47.3 ± 4.8 ^a^ | 2.8 ± 0.5 ^a^ | 1.7 ± 0.3 ^a^ |
| CS:MD |  | 1.2 ± 0.0 ^c^ | 0.2 ± 0.0 ^ab^ | 0.2 ± 0.0 |  | 23.6 ± 1.4 ^ab^ | 0.9 ± 0.0 ^bc^ | 0.5 ± 0.0 ^b^ |
| CS:HD |  | 0.5 ± 0.0 ^c^ | 0.2 ± 0.0 ^b^ | 0.1 ± 0.0 |  | 23.9 ± 3.4 ^ab^ | 2.0 ± 0.1 ^ab^ | 1.3 ± 0.1 ^a^ |
| **p-value** |  | **<0.0001** | **0.0276** | 0.1059 |  | **0.0179** | **0.0007** | **0.0005** |
| HS |  | 2.6 ± 0.0 | 0.2 ± 0.0 | 0.2 ± 0.0 |  |  |  |  |

Results are represented by the mean ± standard deviation of the Supernatant and Precipitate apparent viscosity (η) obtained by three repetitions compared by ANOVA (p<0.05). Different letters in the same column indicate significant differences between the samples at p<0.05 by Tukey's multiple comparisons. Human saliva (HS) is reported separately as a reference fluid and does not correspond to a supernatant or precipitate fraction. The nomenclature refers to the variety, CMR: Carménère, CG: Carignan; CS: Cabernet Sauvignon, and the astringency sub-quality of the drying type, ND: No Drying; LD: Low Drying; MD: Medium Drying; HD: High Drying.

**Figure S-1.** Shear viscosity as a function of shear rate and storage modulus G’ (Pa) (solid symbols) and loss modulus G’’ (Pa) (open symbols) from oscillatory frequency sweeps for supernatant (A-C) and precipitate (B-D) from the mixtures of red wine – human saliva (1:1).

**Figure S-2.** Apparent viscosity of supernatant (left plot) and precipitate (right plot) at shear rates of 1 s^−1^ (η_1_, open symbols), 50 s^−1^ (η_50_, red symbols), and 100 s^−1^ (η_100_, black symbols). The dotted lines represent the viscosity ranges of the IDDSI consistency classification at a shear rate of 50 s^−1^.

**Figure S-3.** Principal Component Analysis (PCA) of the red wines: (A) Carménère-ND, Carignan-LD-HD and Cabernet Sauvignon-LD-MD-HD, (B) Carménère-ND and Carignan-LD-HD, (C) Carménère-ND and Cabernet Sauvignon -LD-MD-HD (D) Carménère -ND and Cabernet Sauvignon -MD -HD, including the physical properties from the mixture with HS (Coefficient of Friction, CoF_w+hs_; Film Thickness, FT_w+hs_; Apparent Viscosity at 50 s^−1^, η_50 precipitate_), and chemical properties (Polysaccharides Content, Poly _precipitate_; Protein Content, Prot _precipitate_; Total Phenol Index, TPI _precipitate_; Total Tannins Content, TTC _precipitate_; Total Anthocyanins Content, TAC _precipitate_).
